# Evaluation of integrated disease surveillance and response (IDSR) core and support functions after the revitalisation of IDSR in Uganda from 2012 to 2016

**DOI:** 10.1186/s12889-018-6336-2

**Published:** 2019-01-09

**Authors:** Ben Masiira, Lydia Nakiire, Christine Kihembo, Edson Katushabe, Nasan Natseri, Immaculate Nabukenya, Innocent Komakech, Issa Makumbi, Okot Charles, Francis Adatu, Miriam Nanyunja, Solomon Fisseha Woldetsadik, Ibrahima Socé Fall, Patrick Tusiime, Alemu Wondimagegnehu, Peter Nsubuga

**Affiliations:** 1 Epidemiology and Surveillance Division, Ministry of Health, P.O Box 7072, Kampala, Uganda; 2 Public Health Emergency Operation Centre, Ministry of Health, Kampala, Uganda; 3 World Health Organization, Uganda Country Office, Kampala, Uganda; 4 World Health Organization Africa Regional Office, Kampala, Uganda; 5 National Disease Control, Ministry of Health, Kampala, Uganda; 6 Global Public Health Solutions Inc, Atlanta, GA USA

**Keywords:** Integrated disease surveillance and response, Core indicators and core functions, Uganda

## Abstract

**Background:**

Uganda is a low income country that continues to experience disease outbreaks caused by emerging and re-emerging diseases such as cholera, meningococcal meningitis, typhoid and viral haemorrhagic fevers. The Integrated Disease Surveillance and Response (IDSR) strategy was adopted by WHO-AFRO in 1998 as a comprehensive strategy to improve disease surveillance and response in WHO Member States in Africa and was adopted in Uganda in 2000. To address persistent inconsistencies and inadequacies in the core and support functions of IDSR, Uganda initiated an IDSR revitalisation programme in 2012. The objective of this evaluation was to assess IDSR core and support functions after implementation of the revitalised IDSR programme.

**Methods:**

The evaluation was a cross-sectional survey that employed mixed quantitative and qualitative methods. We assessed IDSR performance indicators, knowledge acquisition, knowledge retention and level of confidence in performing IDSR tasks among health workers who underwent IDSR training. Qualitative data was collected to guide the interpretation of quantitative findings and to establish a range of views related to IDSR implementation.

**Results:**

Between 2012 and 2016, there was an improvement in completeness of monthly reporting (69 to 100%) and weekly reporting (56 to 78%) and an improvement in timeliness of monthly reporting (59 to 93%) and weekly reporting (40 to 68%) at the national level. The annualised non-polio AFP rate increased from 2.8 in 2012 to 3.7 cases per 100,000 population < 15 years in 2016. The case fatality rate for cholera decreased from 3.2% in 2012 to 2.1% in 2016. All districts received IDSR feedback from the national level. Key IDSR programme challenges included inadequate numbers of trained staff, inadequate funding, irregular supervision and high turnover of trained staff. Recommendations to improve IDSR performance included: improving funding, incorporating IDSR training into pre-service curricula for health workers and strengthening support supervision.

**Conclusion:**

The revitalised IDSR programme in Uganda was associated with improvements in performance. However in 2016, the programme still faced significant challenges and some performance indicators were still below the target. It is important that the documented gains are consolidated and challenges are continuously identified and addressed as they emerge.

## Background

The Integrated Disease Surveillance and Response (IDSR) strategy was adopted by the African Region of the World Health Organisation (WHO-AFRO) in 1998 to serve as a comprehensive strategy to improve disease surveillance and to improve laboratory and response capacities of WHO Member States in Africa [1, 2]. The IDSR strategy was developed in response to a series of emerging and re-emerging diseases which killed large numbers of people in the African region [3–5]. Nearly two decades after the adoption of the IDSR strategy, the relevance of the strategy is becoming even more pronounced as countries face the double burden of communicable and non-communicable diseases [6–8]. The situation for communicable diseases is made worse by the increase in global travel [9–11], in today’s highly interconnected world. The threat posed by international travel with regards to the spread of infectious pathogens was seen as a potential catalyst for the development and adoption of the International Health Regulations (IHR) 1969 by WHO Member States which were revised and replaced with IHR (2005) [12, 13]. The IHR (2005) were expanded to cover both prevention of the international spread of infectious and non-infectious health threats with minimal interference with international travel and trade [5, 13]. Since the IDSR framework and IHR (2005) requirements share common goals, WHO Member States in the African region decided to make use of the IDSR strategy as a platform for implementation of IHR (2005) [5].

The IDSR and IHR frameworks provide an opportunity for low-income countries to leverage their limited resources to continuously improve their disease surveillance and response systems. The effectiveness of a public health surveillance system depends on its ability to adequately monitor priority health events to generate quality and timely information that can be used to initiate appropriate public health actions [14, 15]. WHO recommends regular assessment of IDSR core functions (which include case detection, case confirmation, case registration, case reporting, data management, data analysis, outbreak preparedness, outbreak response and feedback) and support functions (which include guidelines, laboratory capacity, supervision, training, resources and co-ordination) at all levels of the health system [14, 16].

Uganda is one of the low-income countries that remain at risk and continues to experience disease outbreaks caused by emerging and re-emerging diseases such as cholera, meningococcal meningitis, typhoid, and viral haemorrhagic fevers [5, 17–20]. Uganda adopted the IDSR strategy in 2000 and started to implement it in 2001 [21]. Before implementation of the IDSR strategy, a baseline assessment of the existing vertical surveillance systems was conducted whose findings informed the development of a 5-year implementation plan and guided the IDSR implementation process [21, 22]. After several years of IDSR implementation in Uganda, assessment of the performance of the program revealed improvements in IDSR indicators such as timeliness and completeness of reporting, case detection, response to epidemics and training [21, 23]. However, these assessments also highlighted challenges such as the low laboratory capacity for disease confirmation, the decline in government funding, the limited availability of laboratory supplies and the linkage of laboratory and surveillance data [21, 23].

In 2009, 2 years after implementing IHR (2005) within the IDSR framework as agreed by WHO Africa Member States [24], an assessment of progress in improving IHR core capacities was conducted. This assessment also documented successes and challenges including district and national-level inconsistencies and inadequacies in IDSR core activities and support functions such as training, support supervision, communication and feedback [25]. In 2012, the Uganda Ministry of Health (MOH) in collaboration with the WHO Country Office and other key partners implemented a plan to revitalise the IDSR program.

Implementation of the revitalised IDSR program involved scaling up activities related to building capacity at the districts to detect, report and respond, in a timely manner, to public health events. The major activity under this approach was IDSR training which focused on health facility-level health workers, District Task Forces (DTFs) and District Epidemic Preparedness and Response Committees (EPRC). Other activities included operationalization and dissemination of of the revised IDSR guidelines, incorporation of the revised IHR 2005 guidelines into IDSR guidelines, updating of the IDSR training materials and revision and dissemination of IDSR data collection tools.

The Uganda MOH evaluated the revitalised IDSR programme to document whether the programme was implemented adequately. In this study, we present findings from an assessment of IDSR core activities and support functions 5 years after implementation of the revitalised IDSR programme. The information from this evaluation is important to strengthen disease surveillance and to guide the IDSR implementation process not only in Uganda but also in other WHO-AFRO countries with similar settings.

## Methods

### Study setting

Health service delivery in Uganda is organised in tiers; from Health Centre (HC) I, HC II, HC III, HC IV, general hospital, regional referral hospital and national referral hospital. Operationally, HC I are Village Health Teams that provide referral services to higher levels. HC IIs provide basic out-patient and preventive services. HC IIIs provide services offered by HC IIs in addition to in-patient medical and antenatal services and deliveries. HC IVs are points of referral for the lower facilities and provide services offered by HC IIIs in addition to emergency surgeries such as caesarean sections. General hospitals are points of referral for the lower facilities and provide a range of medical, surgical and preventive services. Regional referral hospitals are points of referral for district hospitals and provide specialised medical, surgical, obstetric/gynaecological and paediatric services. National referral hospitals are points of referral for regional referral hospitals and provide highly specialised medical, surgical, obstetric/gynaecological and paediatric services services. Health facilities from HC I to the general hospital are managed by local governments through decentralisation whereas regional and national referral hospitals are semi-autonomous institutions. For effective health service delivery, Uganda is further sub-divided into 14 health regions which are supervised by regional referral hospitals.

### Study design

The evaluation was a cross-sectional survey that employed mixed quantitative and qualitative methods. Qualitative data was collected using Focus Group Discussions (FGDs) and Key Informant (KI) interviews to enable better interpretation of quantitative findings and exploration of a range of different views related to IDSR implementation.

### Sampling and sample size estimation

**Fig. 1:**
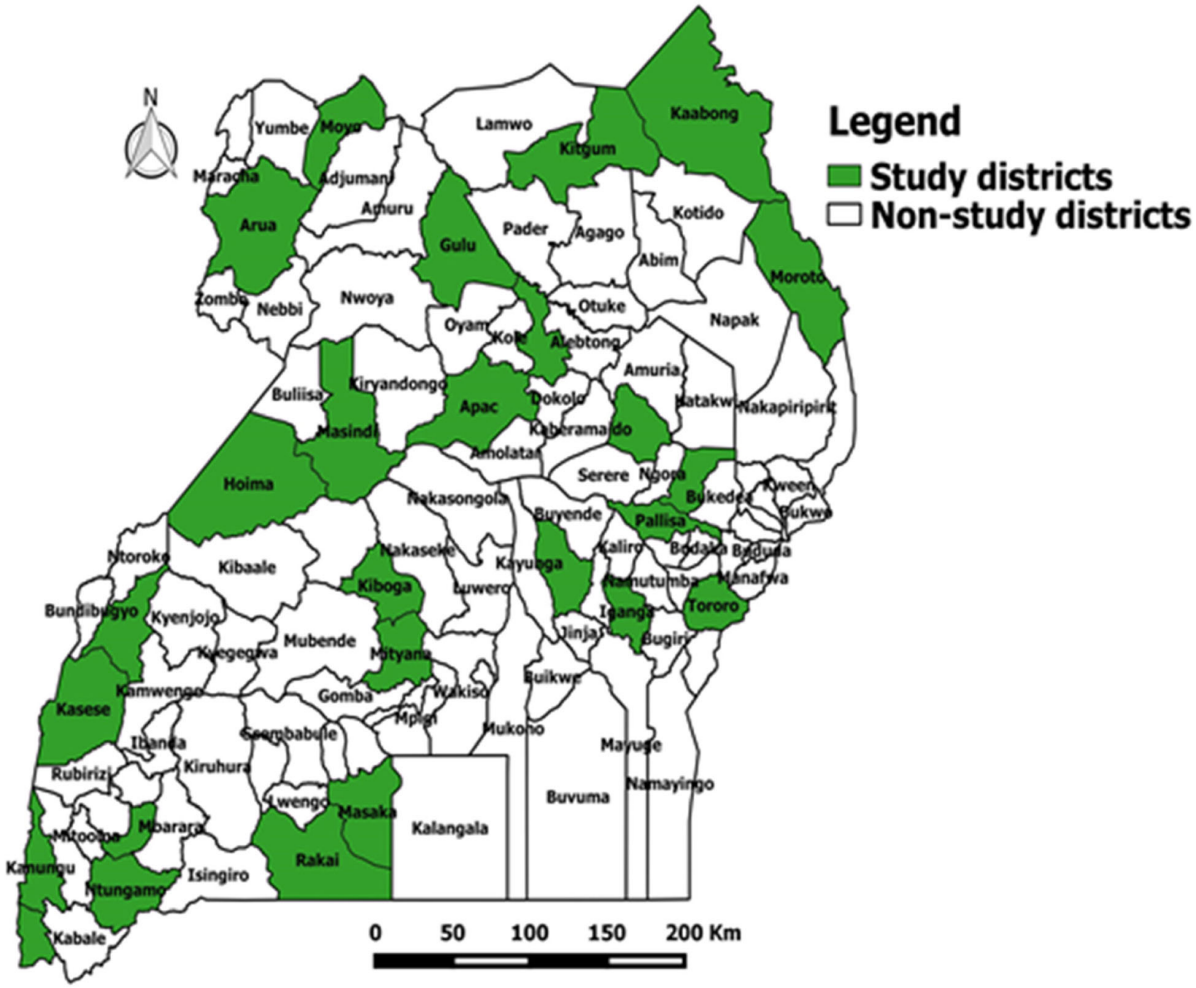
Distribution of districts where IDSR evaluation was conducted (map was created by authors)

We conducted this evaluation in 26 districts (Fig. 1) selected from 13 of the 14 health regions that had completed IDSR training. Two districts were purposively selected from each health region based on presence of a general hospital and a HC IV. For health regions that had more than two districts fulfilling the above criteria, we selected the districts for evaluation using a simple random sampling method. In each of the districts, one general hospital, one HC IV, two HC IIIs and three HC IIs were selected. If a district had more than the required number of eligible health centres, those for evaluation were selected using simple random sampling. A total of 26 general hospitals, 26 health centres level IV, 52 health centres level III and 78 health centres level II were selected (Table 1).

**Table 1:** Characteristics of participants and type of health facilities

Characteristic	Number (%)
Type of health facility (*n* = 202)	
Public	164 (90.1)
Private not for profit	17 (9.3)
Private for profit	1 (0.5)
Health facility level (n = 202)	
Hospital	26 (14.3)
Health centre IV	26 (14.3)
Health centre III	52 (28.6)
Health centre II	78 (42.8)
IDSR knowledge assessment (*n* = 606)	
Clinicians and Nurses	391 (64.5)
Health facility laboratory workers	108 (17.8)
District Health Team	81 (13.4)
District Laboratory Focal Persons	26 (4.3)
Duration after IDSR training (n = 606)	
< 12 months	346 (57.1)
> 12 months	260 (42.9)
FGDs participants (*n* = 216)	
Nurses	101 (46.8)
Clinicians	32 (14.8)
Laboratory	35 (16.2)
Others	48 (22.2)
KI participants (*n* = 32)	
District level	26 (81.3)
National level	6 (18.7)

Using the sampling formula, n = [Z^2^ x p(1 - p)] **/** e^2^, the minimum number of health workers to be interviewed during assessment of IDSR knowledge and confidence in performing key IDSR tasks was 442. This was calculated at Z = 1.96 for 95% confidence level, p (expected true proportion) = 0.5, e (desired precision) = 0.05 and was adjusted for 15% non-response. From each district, we selected at least three health workers belonging to the District Health Team (DHT), at least four health workers from each general hospital (of which at least one was a laboratory worker), at least three health workers from each HC IV (of which one was a laboratory worker), at least seven health workers from HC IIIs (of which one was a laboratory worker) and at at least three health workers from HC IIs. Only health workers who had undergone IDSR training were selected.

FGDs were conducted at general hospitals. Five to ten hospital-based health workers, at least three of which had been trained in IDSR, were selected to participate in each FGD. Interviewers ensured representation from different hospital departments. In-Charges of hospitals were excluded from FGDs to allow for free discussions.

Key informants (KI) were selected from the district and national levels to provide insight into issues related to IDSR implementation. At the district level, 26 District Health Officers (DHO) were selected whereas at the national (MOH) level, six programme managers or heads of departments were selected.

### Data collection

The evaluation team developed quantitative and qualitative data collection tools. These tools were pre-tested in one of the districts which had completed IDSR training. The pre-test was followed by revision and re-testing of the tools before the commencement of the evaluation from 14th June to 1st July 2016. Prior to the evaluation, research assistants were recruited and underwent a five-day training focusing on the objectives and the design of the evaluation and data collection among others. All the interviews were conducted in English.

Quantitative data was collected by administering the following standardised questionnaires and check lists:

i) Health worker IDSR knowledge survey questionnaires: Four different questionnaires were used and these included: a) the health facility level clinicians and nurses b) health facility level laboratory health workers c) the DHT members and d) the district laboratory focal persons. The aim of using these tools was to assess IDSR knowledge and the level of confidence in performing specific IDSR tasks before and after the IDSR training. To assess IDSR knowledge, two standard case scenarios (in the form of questions) were developed from four IDSR modules delivered during the training. These modules were: identification, reporting, investigation/confirmation and analysis/interpretation of data on priority public health events. We assessed the level of confidence in performing 4 key IDSR tasks before and after receiving IDSR training. The IDSR tasks assessed included: a) confidence in the use of standard case definitions b) generating weekly epidemiological reports c) conducting descriptive data analysis and d) determining whether to investigate an outbreak. This assessment was done by asking health workers to grade their level of confidence on a Likert scale (levels 1 to 5). We conducted 391 interviews among clinicians and nurses, 108 among health facility level laboratory health workers, 81 among members of the DHT and 26 among the district laboratory focal persons (Table 1).
ii) IDSR observation check lists: Three separate observation check lists were used to observe and document IDSR performance indicators at national, district and health facility levels. Overall, one check list was filled at national level, 26 at district level, 26 at general hospital level, 26 at HC IV level and 79 at HC III level. Health centres level II were excluded from these observations due to the lack of a functional laboratory.
iii) IDSR training cost analysis tool: We conducted an analysis of the direct costs incurred on trainees by reviewing IDSR training support documents at MOH and extracting the relevant expenditures. These expenditures included per diem (lodging, meals and incidentals) for trainers, trainees and support staff, transport reimbursements, course training materials, fuel and communication.

We collected qualitative information to guide the interpretation of quantitative findings and to establish a range of views related to IDSR implementation that were not captured during the quantitative assessment. The qualitative information was collected using;

i) FGDs: Interviewers were guided by an FGD guide which contained standard IDSR issues for exploring. The responses from participants were captured on paper and audio-tape and the discussion was allowed to go on until no new issues could be elicited from participants. Information collected from FGDs included: a) benefits of IDSR training b) aspects of the training that are not good c) main aspects of IDSR training that were used in day-to-day work d) aspects of IDSR training that participants were not able to practice and d) recommendations to improve the training. A total of 26 FGDs were conducted in which 160 respondents participated. Of these respondents, 101 were nurses, 32 were clinicians, 35 were laboratory workers and 48 were other cadres (Table 1).
ii) KI interviews: We administered a structured questionnaire to 26 district level and 6 central (MOH) level KIs to obtain their views about IDSR implementation (Table 1). Information captured included: a) opinions about the adequacy of IDSR design b) IDSR programme achievements c) challenges faced by the IDSR programme d) mainstreaming of IDSR training e) good practices and lessons learnt and f) suggestions to improve the IDSR programme in Uganda.

In this manuscript, we present qualitative data that can directly explain the quantitative findings. Detailed qualitative findings will be published in another manuscript.

### Data management and analysis

We recorded data both on paper and electronically on Android phones using Magpi software (www.magpi.com). The health region supervisors checked data for completeness after each interview. We selected 10% of the paper-based data questionnaires and entered them into Epi-Info 7 (US Centers for Disease Control and Prevention) and compared them with Magpi data to ensure that there was agreement. Epi-Info was used to analyse data.

We computed IDSR indicators at national, district and health facility levels for the period after revitalisation of IDSR (2012 – 2016) and compared these indicators with those in the period before revitalisation (years 2004 and 2011) and the targets set by the MOH. The 2004 and 2011 indicators were extracted from published assessments [21, 22], reports and the national database (DHIS-2). We computed and tabulated the proportion of health workers who gave a correct response to each IDSR question/scenario. Knowledge retention was assessed by comparing average marks scored by health workers who were trained < 12 months before the evaluation and those trained ≥12 months before the evaluation and significance levels tested using a chi-squared test.

During data analysis, we categorised confidence levels into two main categories; “Not confident” (confidence level 1 - 3) and “Confident” (confidence level 4 & 5) and calculated the proportion of responses in each category before and after the IDSR training. These proportions were then compared using the paired sample t-test. We computed the average cost per IDSR trainee by dividing the total direct costs by the total number of health workers trained.

We used a thematic approach to analyse the qualitative data. Transcripts of FGDs and KI interviews were reviewed by the evaluation team to generate a list of issues and themes arising from each question asked to the participants. We then entered the issues identified from each question into Microsoft Excel 2013 and this data was exported to Epi-Info 7 to generate frequencies.

### Ethics approval and consent to participate

This was an evaluation of a public health program which was requested by the MOH. The evaluation was determined to be non-human subject research according to Uganda’s research guidelines [26]. The MOH national task force on epidemics and public health emergencies approved the evaluation protocol, oversaw the conduct of the evaluation and approved the consent procedures. We obtained verbal consent from each health worker identified for the interview. Prior to the interviews, the potential participants were provided with information about the evaluation and were assured that their participation was voluntary and their refusal would not lead to any consequences. Approval to publish this manuscript was sought from the Director General of Health Services through the Commissioner National Disease Control.

## Results

### IDSR performance indicators before and after revitalisation of the IDSR program

#### Completeness and timeliness of reporting at the national level

The completeness of reporting of monthly eidemiological data reduced from 99% in 2004 to 79% in 2011 and 69% in 2012 (the time of initiation of the re-vitilised IDSR program). Thereafter, the completeness of reporting improved to 100% in 2015 and 2016 (Table 2). The timeliness of reporting of monthly data reduced from 88% in 2004 to 69% in 2011 and 59% in 2012 and this was followed by a gradual improvement to 93% in 2016 (Table 2). A similar pattern was observed for weekly epidemiological data; completeness reduced from 96% in 2004 to 72% in 2011 and 56% in 2012 followed by an increase to 78% in 2016 whereas timeliness reduced from 96% in 2004 to 55% in 2011 and 40% in 2012 followed by an increase to 68% in 2016 (Table 2). These achievements were also reflected in the FGDs where respondents identified improved reporting and timeliness as key benefits of IDSR training (Table 5). The FGD respondents attributed these improvements to increased awareness about disease surveillance that was provided during IDSR training and the roll-out of the mobile telephone text message-based reporting system (m-Track):

*‘Before we were trained in disease surveillance, many of us never took surveillance to be important’ (FGD participant, Moyo Hospital).*

*‘Ever since mTrac was rolled out in the district, reporting has become much easier since we are now able to submit weekly reports by use of mobile telephones’ (FGD participant, Kalisizo Hospital).*

**Table 2:** Key IDSR performance indicators before and after revitalisation of the IDSR program

Indicator	Target	Before		After				
		2004	2011	2012	2013	2014	2015	2016
National level indicators								
Completeness of reporting (%)								
Monthly	80	99.0	79.0	69.0	91.2	97.6	100	100
Weekly	80	96.0	71.5	56.2	61.0	51.2	68.8	78.0
Timeliness of reporting (%)								
Monthly	80	88.0	69.1	58.9	73.1	82.0	86.5	92.5
Weekly	80	96.0	55.0	40.3	42.1	50.0	57.8	68.3
Cholera case fatality rate (%)	< 1	2.4	2.2	3.2	3.6	2.8	2.3	2.1
Annualized Non-Polio AFP Rate (per 100,000 population below 15 years)	≥4.0^a^	2.2	2.6	2.8	2.8	3.2	3.3	3.7
Sent feedback to district level (%)	100	100	–	–	–	–	–	100.0
District level indicators								
Analysed data on priority diseases (%)	100	75.0	–	–	–	–	–	61.5
Suspected outbreaks notified to MoH within 48 h (%)	100	46.0	–	–	–	–	–	57.7
Laboratory confirmation for the most recent outbreak (%)	100	61.0	–	–	–	–	–	73.1
District Surveillance Focal Person (%)	100	–	–	–	–	–	–	96.2
District Laboratory Focal Person (%)	100	–	–	–	–	–	–	100.0
Functional epidemic preparedness and response committee (%)	100	65.0	–	–	–	–	–	69.2
Sent feed back to lower levels (%)	100	55.0	–	–	–	–	–	86.6

^a^This is a target adopted by Uganda

#### Cholera case fatality rate, non-polio AFP rate and feedback at the national level

We assessed the case fatality rate of cholera and found that the national target of < 1% was not achieved. Nevertheless, following an initial increase from 2.4% in 2004 to 3.2% in 2012, the cholera case fatality rate reduced to 2.1% in 2016 (Table 2). The non-polio AFP rate (cases per 100,000 population below 15 years) increased from 2.2 in 2004 to 2.6 in 2011, to 2.8 in 2012 and to 3.7 in 2016 which was slightly below the operational national target of ≥4.0 but above the WHO target of ≥2.0 set for countries in the WHO-AFRO region. All (100%) the districts where the evaluation was conducted reported receiving feedback from the national level before and after the revitalization of the IDSR program; the commonest form of feedback being the weekly epidemiological bulletin.

#### District level IDSR performance indicators

We compared 2016 IDSR indicators with the targets set by the MOH and found that targets were not reached for all the indicators assessed (Table 2). By comparing the 2016 IDSR indicators with the 2004 indicators, we noted a) an improvement in outbreak notification to MoH from 46 to 58% b) an improvement in laboratory confirmation for outbreaks from 61 to 73% c) the presence of a functional epidemic preparedness and response committee from 65 to 69% and d) provision of feedback to health facilities from 55 to 87%.

#### Health facility level IDSR performance indicators

Health facility indicators were assessed by observing the presence of key resources for IDSR implementation and evidence of data analysis (Table 3). We found that the availability of standard case definition materials improved from 40% in 2004 to 60% in 2016. Data analysis on at least one priority disease improved from 47% in 2004 to 54% in 2016. Participants of FGDs attributed improvement in data analysis to the IDSR training:

*‘and during the training, a lot of emphasis was put on data analysis, and facilitators ensured that all participants are empowered with basic skills to conduct surveillance data analysis’ (FGD participant, Itojo Hospital).*

Up to 98% of health facilities had outpatient registers for case documentation. Availability of weekly and monthly Health Management Information System (HMIS) data reporting tools slightly improved from 66% in 2004 to 68% during the 2016 evaluation. Health Management Information Officers were available at 60% of health facilities. Tools for notification of measles and acute flaccid paralysis were available at over 90% of health facilities. The cholera specimen transport medium (Cary-Blair) was observed at only 8% of health facilities.

**Table 3:** Health facility IDSR performance indicators; 2016 evaluation vs. 2004 survey [21]

Indicator	Performance 2004 (%)	Performance 2016 (%)
Standard case definitions^a^	40.0	60.4
Patient registers	98.2	98.5
Data analysis on at least one priority disease	47.0	54.0
HMIS data reporting tools	86.0	88.1
Measles case investigation forms	–	90.1
AFP case investigation forms	–	90.1
Health Management Information Officer	–	59.9
Availability of Cary-Blair transport medium for cholera	–	8.4
Received feedback from the district	15	86.6

^a^Case definition materials were observed at 81% of district headquarters

#### IDSR knowledge, knowledge retention and confidence in performing key IDSR tasks

**Fig. 2:**
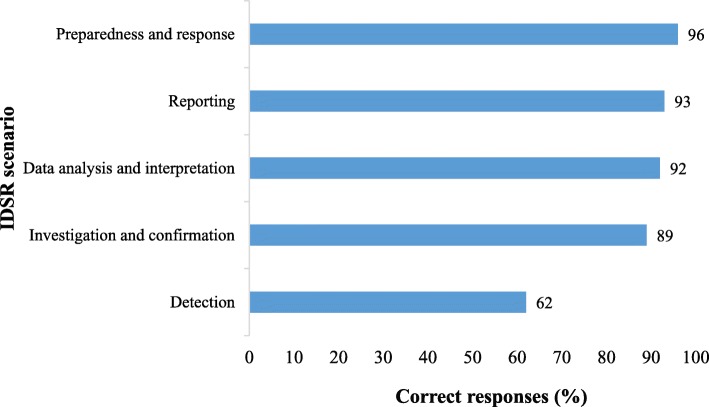
Assessment of IDSR knowledge among health workers at district and health facility levels

We found that health workers at district and health facility levels had good knowledge about IDSR with 86% of them correctly answering all IDSR scenarios. When disaggregated by IDSR scenario, health workers correctly answered; 96% of scenarios on preparedness and response, 93% of scenarios on reporting, 92% of scenarios on data analysis and interpretation, 89% of scenarios on investigation and confirmation and 62% of scenarios on case detection (Fig. 2). There was no statistical difference in the average marks scored by health workers trained < 12 months and those trained ≥12 months (Table 4). Health workers were significantly more confident to perform key IDSR tasks after receiving IDSR training than before receiving the training (Table 5). The average cost incurred per trainee was $217.

**Table 4:** Assessment of IDSR knowledge retention (decay) among health workers

Category health worker	Average mark scored (%)		*P*-value
	Trained < 12 months	Trained ≥12 months	
District health team (*n* = 81)	91.2	89.3	0.78
District laboratory focal persons (*n* = 26)	89.8	85.4	0.75
Health facility level laboratory workers (108)	87.0	88.5	0.85
Health facility level health workers (*n* = 391)	78.9	77.1	0.70

**Table 5:** Assessment of confidence health workers’ levels in performing key IDSR tasks before and after the training

IDSR task	% laboratory workers confident (*n* = 108)			% other health workers confident (*n* = 370)		
	Before	After	*P*-value	Before	After	*P*-value
Using SCDs^a^	4.60 (4.52 –4.74)	72.23 (72.31 – 72.31)	< 0.001	10.33 (10.30 – 10.36)	81.56 (81.52 – 81.60)	< 0.001
Generating HMIS reports	29.62 (29.55 – 29.69)	82.40 (82.32 – 82.47)	< 0.001	23.81 (23.77 – 23.85)	86.49 (86.38 – 86.60)	< 0.001
Data analysis	14.82 (14.65 – 14.99)	65.69 (65.60 – 66.59)	< 0.001	11.94 (11.91 – 11.97)	77.03 (36.99-37.07)	< 0.001
Outbreak investigation	7.59 (7.36 – 7.82)	74.09 (74.01 – 74.17)	< 0.001	13.04 (13.01 – 13.07)	78.94 (78.90-78.98)	< 0.001
IPC^b^	33.30 (33.21 – 33.39)	87.00 (86.94 – 87.06)	< 0.001	41.22 (41.17 – 41.27)	92.89 (92.86-92.92)	< 0.001

^a^SCD: standard case definintion. ^b^ Infection prevention and control

IPC?

Capacity building and mentorship on IDSR provided by the MOH, was pointed out by respondents during FGDs as a major driver for improved confidence in performing key IDSR tasks (Table 6):

‘after the training, our attitude towards surveillance completely changed and we are now able to compile and submit all our weekly and monthly reports to the district without fail’ (FGD participant, Moyo Hospital)*.*

*‘A few months ago, we had a yellow fever outbreak in our district which affected many people, but fortunately, this came at a time when the ministry of health had built our capacity in responding to disease outbreaks. We were able to investigate and respond to this outbreak’ (FGD participant, Masaka Hospital).*

*‘whenever there was an epidemic, doctors would come from the MOH headquarters to handle the situation, but nowadays the district is much more involved. We were trained on how to handle disease outbreaks and surveillance’ (FGD participant, Gulu Hospital).*

**Table 6:** Issues related to IDSR implementation identified during qualitative interviews

IDSR Aspect	Major issues
1.0 Achievements of IDSR training^a^	1.1 Completeness of reporting has improved
	1.2 Timeliness of reporting has improved
	1.3 Improvement in case detection
	1.4 There is better response to outbreaks
	1.5 Data analysis has improved
2.0 Reasons for improved confidence in executing IDSR tasks^a^	2.1 Capacity building trainings
	2.2 Supervision and mentorship
3.0Challenges affecting IDSR implementation^b^	3.1 Inadequate number of trained health workers
	3.2 Inadequate funding
	3.3 Some health workers perceive IDSR to be vertical program
	3.4 Irregular supervision
	3.5 High turnover of trained health workers
4.0 Recommendations to improve future IDSR training^b^	4.1 Train more health workers
	4.2 IDSR training should be conducted regularly
	4.3 Train community members in IDSR
	4.4 Integrating IDSR into pre-service training
	4.5 Strengthening IDSR support supervision

^a^Data was collected from Focus Group Discussions. ^b^ Data was collected from Key Informant interviews

#### Challenges and recommendations about the revitalised IDSR programme

Key Informants reported that although the revitalized IDSR programme had been successful, some key challenges still existed. These included: a) an inadequate number of trained frontline health workers b) inadequate funding to support IDSR activities at district and health facility levels c) the perception of IDSR as a vertical programme by some health workers d) irregular supervision and e) a high turnover of trained health workers (Table 6).

The Key Informants recommended increasing IDSR funding at district and health facility levels, training of more health workers, incorporating IDSR training into the pre-service curriculum for health workers and strengthening supervision as strategies to improve IDSR performance (Table 6).

## Discussion

This evaluation provided an overview of the performance of the revitalised IDSR core and support functions in Uganda at national, district and health facility levels. Results of the evaluation showed positive changes in IDSR indicators at different levels of the health system, including, reporting and investigation of priority diseases, the cholera case fatality rate, data analysis and feedback. The evaluation showed that health workers had good knowledge of IDSR. There was no evidence of the loss of IDSR knowledge over time and health workers reported more confidence in performing key IDSR tasks after the training.

Improvements in IDSR indicators such as completeness and timeliness of reporting after scaling up IDSR have been observed in other studies in Uganda and elsewhere [21, 27–29]. These improvements may be attributed to a better appreciation of the importance of disease surveillance by health workers after IDSR training and the introduction of an electronic reporting system (DHIS-2) as observed by FGD respondents. Since 2012, monthly epidemiological data is collected from health facilities and submitted to the district biostatistician who loads the data into the DHIS-2 database. Previously, hard-copy reports were physically delivered to the MOH. The introduction of the Short Message Service (SMS) for reporting weekly epidemiologic data (mTrac) has proved to be a powerful tool that empowers health workers and removes many of the barriers associated with paper-based reporting. The mTrac platform is incorporated within the DHIS-2 database to enable weekly epidemiological data reporting from health facility to the national level, using coded mobile telephone SMS. Our findings concerning SMS reporting are similar to those from a study conducted in Tanzania which documented improved completeness and timeliness of disease reporting from 50 to 89% after implementation of an SMS based reporting system [30].

Improved performance on IDSR indicators also reflects good understanding of IDSR among health workers, good feedback from the national to the district level and on to the health facility level and the wide availability of HMIS reporting tools that were observed in this study. In studies conducted in Tanzania and Nigeria, training of health workers on disease surveillance was followed by improvements in IDSR indicators such as reporting [28, 31]. Similarly, two other studies in Kenya and Nigeria documented that the availability of appropriate tools led to improvements in reporting of surveillance data from health facilities [32, 33].

We found that the annualised non-polio AFP rate (NPAR) improved over time and was found to be consistently higher than the target set by WHO-AFRO. This performance is sensitive enough to detect cases of wild poliovirus in Uganda. Annualized NPAR reported from our evaluation are higher than what was reported from studies in South Africa and Ghana [34, 35] but lower than what was reported from Nigeria [36]. It is important that Uganda’s achievements in polio surveillance be sustained since global efforts towards polio eradication identified AFP surveillance as an essential component of the eradication strategy [37].

Feedback from higher to lower level health facilities is a major motivating factor among health workers and may have played a significant role in the improvement of key IDSR indicators such as reporting. In this study, we found that all districts and most of the health facilities received feedback from a higher level facility. Feedback from national to district level was provided in the form of dissemination of the MOH weekly epidemiological bulletin, support supervision visits and telephone calls while health facilities received feedback mainly from support supervision visits, from direct telephone calls from national and district levels and from quarterly review meetings. The feedback from district to health facilities observed in this study was 87% compared to 15% in 2000 and 55% in 2004 [21, 22].

Another strategy that may have enhanced IDSR performance was the involvement of the leadership at different levels in the IDSR training. Training was organised in such a way that leaders received a one-day IDSR training. However, it is unclear how this training may have impacted on the performance of IDSR and further studies are required. Additionally, there is a need to identify gaps in this critical area, as the district leadership for example, is expected to provide oversight for the implementation of the IDSR programme and guidance during outbreaks and other public health emergencies.

Although there were improvements in IDSR performance indicators compared to earlier periods, we noted that some indicators were still below the target, especially at the district level. It has been argued that the creation of new administrative units, such as districts, might be one of the major factors responsible for poor performance because these new units often lack sufficient technical capacity to implement IDSR [21]. The number of districts in Uganda increased from 112 at the beginning of the revitalised IDSR programme to 116 in 2016 when this study was conducted. Other important factors that might be responsible for poor performance are the lack of adequate numbers of trained staff and the high attrition rate of staff trained in IDSR, both of which were documented during the qualitative interviews. Previous studies conducted in different settings have also linked poor IDSR performance to lack of adequately trained personnel [28, 38–40].

However, it should be noted that understanding of the dynamics and factors affecting IDSR performance is complex. Nevertheless, it is essential that countries identify and appreciate the bottlenecks in IDSR performance and identify feasible country-specific solutions. In broad terms, enhancing IDSR performance requires efficient support activities that include leadership, communication, training, supervision and the provision of key resources [41]. Strenthening the IDSR support activities requires strong multi-sectoral collaboration and inputs from stakeholders at all levels within the health sector. During the implementation of the revitalised IDSR programme in Uganda, several partners provided support to the MOH which led coordination, implementation and supervision of IDSR activities. Many FGD respondents linked IDSR training to the observed improvements in IDSR indicators. However, they also noted that IDSR support supervision is still irregular at the health facility level. Irregular IDSR supervision has previously been highlighted as a key challenge affecting public health surveillance in other studies [25, 27].

Further improvements in IDSR performance require the consolidation of current gains as well as the introduction of innovative ways to further strengthen the surveillance and response system in Uganda. A multi-sectoral approach should be used where all stakeholders pursue a common strategic goal of developing a workforce that can support public health surveillance and response. Workforce development requires empowering animal and human health workers with the necessary skills in public health surveillance and response; through training, retraining, mentoring and supervision. In-service training was a key intervention to improve IDSR performance during the implementation of the revitalised IDSR program in Uganda. However, the average cost of $217 per trainee that was spent during IDSR training was high and difficult to sustain in the long term. It is therefore critical that the MOH and development partners find innovative, cost-effective and sustainable strategies for improving IDSR awareness and knowledge among health workers. Strategies to consider include the incorporation of IDSR training into pre-service curricula for all health workers which, if implemented, can effectively address gaps related to the apparent lack of resources for training in IDSR competence.

The main strength of this study was the use of mixed quantitative and qualitative methods. This approach provided a platform for gaining a deeper and broader understanding of the different IDSR issues assessed. The major limitation of this study is that the evaluated districts were not randomly selected and therefore our findings may not be generalisable to the entire country. This study does however provide a comprehensive overview of how the revitalised IDSR program has impacted on IDSR performance and the challenges that need to be addressed.

## Conclusions

The revitalised IDSR programme was associated with improvements in IDSR performance at national, district and health facility levels. However, the programme still faces significant challenges with some performance indicators that are still below target. Improved performance requires consolidating current gains, strong collaboration from all stakeholders, supervision and regular review of performance to identify and address challenges as they emerge.

## Abbreviations

DHIS-2: District Health Information Software Version – 2
DHO: District Health Officer
DHT: District Health Team
DTF: District Task Forces
EPRC: Epidemic preparedness and response committee
FGD: Focus Group Discussion
HC: Health Centre
IDSR: Integrated Disease Surveillance and Response
IHR: International Health Regulations
KI: Key informant
NCDs: Non communicable diseases
NPAR: non-polio AFP rate
WHO: World Health Organization
